# Understanding the need for a whole-of-society approach in school nutrition policy implementation: a qualitative analysis

**DOI:** 10.1186/s43058-021-00184-z

**Published:** 2021-07-17

**Authors:** Natasha P. Sobers, Lisa Bishop, Shu Wen Ng, Suzanne Soares-Wynter, Natalie S. Greaves, Madhuvanti M. Murphy

**Affiliations:** 1 George Alleyne Chronic Disease Research Unit, Caribbean Institute for Health Research, University of the West Indies, Cave Hill, Barbados; 2 Gillings School of Public Health, University of North Carolina at Chapel Hill, Chapel Hill, USA; 3 Tropical Metabolism Research Unit, Caribbean Institute for Health Research, University of the West Indies, Mona, Kingston, Jamaica; 4 Faculty of Medical Sciences, The University of the West Indies, Cave Hill, Barbados

**Keywords:** School nutrition policy, Caribbean, Small populations, Healthy eating, Pre-implementation study, Childhood obesity

## Abstract

**Background:**

Only three of twenty Caribbean Community (CARCICOM) countries have mandatory school nutrition policies despite one third of the region’s children being overweight or obese. In Barbados, there are nutrition guidelines which lack the legal mandate of a formal policy. We aim to assess the comprehensiveness of current national nutrition guidelines and to understand the factors operating in the inner and outer school setting that may influence the implementation of a mandatory school nutrition policy from the perspectives of school administrators.

**Methods:**

A documentary analysis of existing nutritional guidelines was conducted along with qualitative semi-structured interviews in primary (elementary) and secondary (high) schools in Barbados. We purposively sampled six primary and four secondary school administrators (principals, deputy principals or senior teachers) to explore their knowledge and views on the National School Nutrition Guidelines. The deterministic implementation paradigm, Consolidated Framework for Implementation (CFIR), was used to explore the complexities within the inner and outer settings of schools. Documentary analysis used a theory-based framework informed by the Wellness School Assessment Tool—school policy analysis questionnaire. Interview transcripts were team coded using thematic analysis with constant comparison facilitated by NVIVO software version12.

**Results:**

School administrators were unaware of the existing National School Nutrition Guidelines which documentary analysis found to be restrictive and weak for implementation. Administrators envisioned a government-led (outer setting), whole of society approach as the most effective strategy for the development and implementation of a proposed mandatory school nutrition policy. School administrators identified lack of financial and human resources as barriers to nutrition policy implementation. Formal and informal food vendors are institutionalized in schools and are influential determinants of the school food environment. Schools have individually reached into the outer setting to work with civil society organizations and private individuals to provide financial support and nutrition expertise to their institutions. Mass media campaigns in the outer setting may influence child and parental food choices.

**Conclusion:**

School administrators describe that government-led, CSO supported policy development using a whole-of-society approach has implications for improving nutrition policy implementation. Our findings demonstrate the use of a deterministic implementation framework in the pre-implementation phase of school nutrition policy development.

**Supplementary Information:**

The online version contains supplementary material available at 10.1186/s43058-021-00184-z.

Contributions to the literature

- A government-led approach is perceived as more effective in facilitating the development of a mandatory school nutrition policy than individual schools devising internally derived healthy school initiatives without guidance.
- Using a deterministic implementation framework in the pre-implementation phase facilitated the translation of themes into recommendations that could assist the development of a school nutrition policy.
- In this small island state, civil society organizations have positively influenced the policy environment and implementation capacity around healthy school initiatives.

## Introduction

There is growing recognition of the increasing burden of childhood obesity worldwide and particularly in small island developing states (SIDS) [1, 2]. In the Caribbean and Pacific Islands childhood obesity rates are among the highest globally [3, 4]. In Barbados (an island in the Caribbean), over 30% of older children (9–11 years) and adolescents (13–15 years) are either overweight or obese [5]. These rates are similar to what exists throughout the Caribbean [6, 7]. Several studies have reported that increasing trends in childhood obesity could be addressed through modification of the school environment [8–10] and the 2020 Global Nutrition report recommends the implementation of evidenced-based food policies to guide the nutrition environment of schools [11].

In the Caribbean, the concept of addressing obesogenicity through examination of the school environment and implementation of school-based interventions is a topic currently being discussed and with interventions being implemented in some countries. Data compiled by the Healthy Caribbean Coalition (HCC) indicates that of the twenty Caribbean Community (CARICOM) member states, five (Bahamas, Barbados, Bermuda, Grenada, Haiti) have national school nutrition policies or guidelines implemented in schools with an additional five (Cayman Islands, Dominica, Guyana, Jamaica, Trinidad and Tobago) under development or partially implemented [12].

In 2015, the National Nutrition Centre of Barbados released guidelines aimed at improving the health of children and adolescents through a focus on the school environment. These guidelines lack a legal framework for enforcement and thus advocates are seeking to open a policy window to facilitate the development of a mandatory school nutrition policy (MSNP) [13]. In this paper, we define nutrition policy as a statement made by government (authoritative body) which provides a mandate for a healthy school environment backed by a framework for enforcement. We consider nutrition guidelines to be statements made with a similar aim but lacking a legal mandate. Local and regional advocacy groups such as the Heart and Stroke Foundation of Barbados (HSFB) and HCC have intensified their calls for a nutrition policy that restricts the sale and marketing of unhealthy foods and drinks in schools.

We aim to assess the depth of adoption and comprehensiveness of current national nutrition guidelines and to explore the settings under which a MSNP will be developed and implemented. We chose to use aspects of the deterministic framework known as the Consolidated Framework for Implementation Research (CFIR) [14] to inform our understanding of the implementation process around school nutrition policies in this Caribbean nation.

## Methods

### Setting

Barbados, a small island developing state in the Caribbean (166 square miles), has 97 primary schools (68 public; 25 private) and 30 secondary schools (23 public; 7 government-assisted private) which include children ages 3–11 and 11–18 respectively. All government primary (elementary) schools participate in the free lunch programme [15]. Students in public secondary (high) school who demonstrate financial need also receive free lunches. Students in private primary schools and the vast majority of secondary school students are expected to either bring lunch from home or purchase from an onsite school canteen. Students are generally not permitted to leave the campus during school hours.

### Documentary analysis

We performed a qualitative enquiry using triangulation methods of documentary analysis and in-depth interviews. We began with a documentary analysis of the nutritional guidelines using a theory based framework informed by a modified version of the Wellness School Assessment Tool (WellSAT) questionnaire [16]. The theory was applied using content-based analytics. The WellSAT tool has high feasibility and relevance to policy evaluation [16]. Performing documentary analysis before the later interviews allowed comparisons to be made between actual and perceived content.

We assessed the comprehensiveness and strength of three government produced documents aimed at facilitating a healthy school environment. These documents are as follows: (1) National Plan of Action for Childhood Obesity Prevention and Control (2015-2018): Barbados Childhood Obesity Prevention Program (B-CHOPP); (2) Nutrition and Healthy Foods in Schools: Nutritional and Practical Guidelines for Barbados (NSNG); and (3) Nutritious and Healthy Foods in Schools: Guidelines for Canteen Operators. We used a modified version of the *WellSAT Score Sheet* [17] containing four sections and 40 items which has been used in school policy analysis in previous studies [18–20]. The WellSAT questionnaire is based on assessing how well the nutrition policy/guidelines address the following domains: *Nutrition Education; Nutrition Standards for Food and Beverages Provided and Sold (in schools)*; *Promoting Healthy Food and Nutrition Environment*; and *Communication and Evaluation*.

Each of the 40 items on the WellSAT was scored as either 0 (not mentioned), 1 (mentioned but recommendation/statement was weak), or 2 (strong statement or recommendation made). Each of the four sections was scored according to comprehensiveness and strength. Comprehensiveness was measured by counting the number of items rated as “1” or “2”, dividing by the number of items in the section and multiplying by 100 to produce a percentage. Strength is calculated by counting the number of items rated as “2”, dividing by the number of items in the section and multiplying by 100. These calculations are in accordance with previously validated usage.

### School level in-depth interviews

We purposively selected administrators from six primary and four secondary co-educational schools with representation from a rural and an urban school, primary and secondary setting and privately and publicly funded institutions. Semi-structured interviews were conducted with school administrators (principals or deputy principals/senior teachers) and data collected using a CFIR informed interview guide (Additional file 1). We focused on the following constructs of the CFIR framework: knowledge, adaptability, complexity and adoption of guidelines; implementation planning; and costs of implementation.

Interviews with administrators were conducted by LB, audio-recorded and transcribed verbatim. Participants were telephoned before the interview and informed of the reason for the study using the participant information sheet (Additional file 2). Written consent was attained before the start of the face-to-face interviews, which were conducted at each administrator’s school. LB was not personally acquainted with any of the participants before making the initial call to schedule the interview. Interviews lasted approximately 25 to 55 min and were sometimes interrupted by normal school proceedings.

A hybrid coding mechanism was conducted by developing a deductive coding dictionary based on the CFIR framework, but also allowing for further codes to inductively emerge from the data. Three broad (parent) nodes/themes from CFIR were initially explored: (1) characteristics of the intervention, (2) inner setting and (3) outer setting. Several child nodes emerged from each of these broader themes. Thematic content analysis with constant comparison across 10 participants in 10 schools was treated as a single-case study across Barbados. From the three CFIR domains of interest, we present the constructs that were found as themes in content analysis (Additional file 3 Supplemental Table 1).

The analysis team had a strong grounding in qualitative research; LB is a female qualitative researcher with a personal interest in childhood obesity, NS is a female post-doctoral researcher with a strong positivist approach and MM, the lead qualitative methodologist on the team, is a female with a doctorate in public health and over 15 years’ experience in qualitative work. The transcripts were double coded (LB and NS) with interrater reliability quickly established after the first two interviews, potentially due to the similarity in research interests and backgrounds of the analysts. Because of the small sample size and to ensure high data quality, we chose to double code 100% of transcripts.

Field notes were reviewed in team discussions involving NS, LB and MM after the first two interviews and again after interview number eight. After eight interviews, it was determined that further data was needed from at least on other private school and one additional public secondary school to ensure that saturation had been reached. After the tenth school interview, the researchers were satisfied that saturation had been reached, and no further interviews were conducted.

Permission to conduct this study was obtained from the Institutional Review Board of the University of the West Indies/Ministry of Health and Wellness. Further permission was obtained from the Ministry of Education to enter school premises to interview key school administrators. We followed the Consolidated criteria for reporting qualitative research guidelines [21] in reporting on this study and have completed the appropriate checklist (Additional file 4).

## Results

### Documentary analysis of existing guidelines

We first sought to understand the scope of the existing national guidelines. The National Plan of Action for Childhood Obesity Prevention and Control (2015–2018): Barbados Childhood Obesity Prevention Program (B-CHOPP) is a high-level document whose scope encompasses a national approach to childhood obesity. While school initiatives are mentioned, detailed strategies are missing. It was noted that specific school level guidelines were being developed. The Nutrition and Healthy Foods in Schools: Nutritional and Practical Guidelines for Barbados (NSNG) developed predominantly by the Barbados Ministry of Health and Wellness is intended to guide school nutrition activities in all schools in Barbados. Its focus is on student nutrition to the exclusion of physical activity and does not focus on actions of teachers or ancillary staff. In fact, the word “teacher” only appears once, in reference to Parent Teacher’s Associations as stakeholders in children’s healthy eating. There is no mention of Civil Society involvement. Finally, the “Nutritious and Healthy Foods in Schools: Guidelines for Canteen Operators” is a sub-document of the NSNG. Because it had a targeted audience, it only addresses issues within the remit of sanctioned Canteen Operators.

Using the WellSAT Evaluation Tool, the current Barbadian policy documents only covered the domain of *Nutrition Standards for Food and Beverages Provided and Sold (in schools)* and *Promoting Health Food and Nutrition Environment.* These guidelines were found to be moderately comprehensive while the recommendations were considered weak (Table 1).

**Table 1:** Analysis of existing national school guidelines

Document description	WELLSAT Domains	Comprehensive score (%)	Strength score (%)
**National Plan of Action for Childhood Obesity Prevention and Control (2015-2018): Barbados Childhood Obesity Prevention Program (B-CHOPP)**	*Nutrition Education*	40	20
	*Nutrition Standards for Food and Beverages Provided and Sold*	77	23
	*Promoting Healthy Food and Nutrition Environment*	7	7
	*Communication and Evaluation*	0	0
**Nutritious & Healthy Foods in Schools: Nutritional and Practical Guidelines for Barbados**	*Nutrition Education*	0	0
	*Nutrition Standards for Food and Beverages Provided and Sold*	85	62
	*Promoting Healthy Food and Nutrition Environment*	29	21
	*Communication and Evaluation*	25	12.5
**Nutritious & Healthy Foods in Schools: Guidelines for Canteen Operators**	*Nutrition Education*	0	0
	*Nutrition Standards for Food and Beverages Provided and Sold*	62	8
	*Promoting Healthy Food and Nutrition Environment*	0	0
	*Communication and Evaluation*	0	0

### Development of mandatory school nutrition policy (MSNP)

**Fig. 1:**
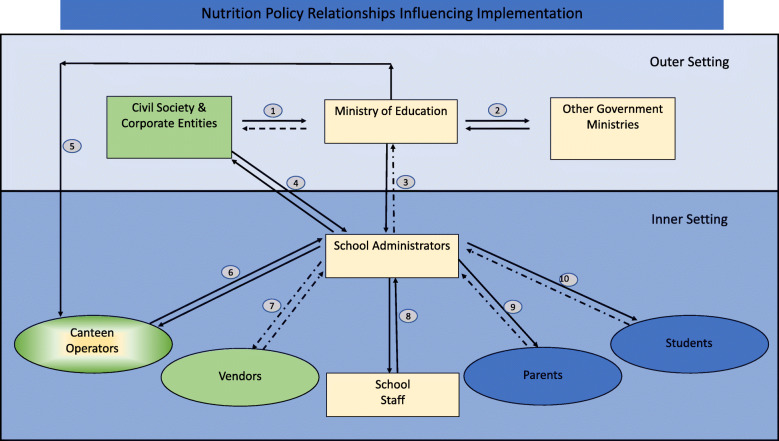
Illustration of direction and quality of information flow. re: School Nutritional Policies

The school administrators were generally unaware of the existence and content of the National guidelines referenced in the documentary analysis. We therefore used the constructs of the CFIR framework which emerged from thematic analysis to discuss administrators’ perceptions on a proposed mandatory school nutrition policy. Administrators provided insights on the source, complexity and opportunity costs of the MSNP and described the inner and outer settings which could influence the future policy intervention (Fig. 1).

### Characteristics of the proposed mandatory school nutrition policy

#### Intervention source

Many participants were keen to point out that mitigation of childhood obesity and premature NCDs needed a high level of buy-in throughout the society and cannot be limited to school. They envisioned a “whole-of- society approach”, referred to the problem of childhood obesity as requiring “national awareness” and that the solution needed to be a “national objective”. School administrators strongly supported an overarching nutrition policy which originated from Ministries of Education, Technological and Vocational Training (METVT) and Health and Wellness (MHW) as opposed to individual-level school policies.

PS3: It’s a greater societal problem yeah. Coming into the environment called school, in my opinion will not be able to make that kind of change because the children go back into the (external) environment. So it has to be a wholistic society, from the time they use the polyclinic when you go for your prenatal discussions and so on, it is everything.

SS2: Schools can only do so much, and I believe there must be a national drive…….. there must be something that is being done by the nation to speak to having healthy lifestyle practices.

SS4: The Law says the Minister (of Education) gives a policy and the board will give me direction on those things. I’m waiting to get the direction and policies on those things.

#### Complexity

The content of a school nutrition policy was thought to require the input of nutrition-trained specialists that are not prevalent within the inner school settings. Primary schools do not have staff trained in nutrition while secondary school teachers may have one per school. Individual activities by schools have therefore been informed by the general health literacy of champions within the school.

#### Cost

School administrators reported that the time and financial costs of investing in a policy were worthwhile for the country given the high levels of childhood obesity but they noted additional costs, that of educational costs to students. The additional curriculum and physical education programming needed to meet the mandate of a nutritional policy could adversely affect the school’s ability to achieve its core mission—classical education in core subjects like Mathematics and English.

### Inner setting

The interviewees perceived the inner setting as having the greatest impact on the success of a future MSNP. Below we describe CFIR constructs related to structural characteristics, culture, networks and communication and implementation climate.

#### Structural characteristics in the inner setting

All public primary schools were part of the national school feeding programme (school meals) and all operated shops run by school staff (school-run shop) (Table 2). Canteens are businesses leased and operated by external food contractors and these were a feature of private primary schools and government secondary schools. Vendors located on or close to the school location as well as food/meals brought to school by students from home are also considered part of the inner setting.

**Table 2:** Characteristics of participating schools

Participant No.	Student age	Location	Food points on campus				
			School meals^a^	School-run shop	Canteen	Vendors	Vending machines
Government primary schools							
PS1	3–11	Urban	✔	✔	NA	✔	X
PS2	3–11	Rural	✔	✔	NA	X	X
PS3	4–11	Rural	✔	✔	NA	✔	X
PS4	3–11	Rural	✔	✔	NA	✔	✔
Private primary schools							
PPS1	3–11	Urban	NA	X	✔	X	X
PPS2	4–11	Urban	NA	X	✔	X	X
Government secondary schools							
SS1	11–18	Urban	NA	X	✔	✔	X
SS2	11–19	Rural	NA	✔	✔	✔	✔
SS3	11–19	Urban	NA	✔	✔	✔	X
SS4	11–19	Rural	NA	X	✔	✔	X

^a^National free school feeding programme; *PS* public primary school, *SS* secondary school, *PPS* private primary school, *✔* in operation, *X not* in operation, *NA* facility not available

#### Culture of the inner setting

The following areas related to the culture of the inner setting were perceived by administrators to be particularly influential.

##### Food habits

Participants expressed that policies or programming would need to encompass all parties within the school community, noting that bad dietary habits were not limited to students, but extended to teachers and parents after whom students model behaviour and who influence and assist with their food choices. The acceptance of unhealthy snacking by everyone was seen as one of these bad dietary habits:

PS2: Students, teachers, parents. Everybody! ‘Cause some teachers in some schools are guilty of eating the snacks along with the children too! (Laugh). Don’t forget we having a lot of influx of younger teachers………..and they would have come up in the system [of unhealthy snacking]. Like me now, we didn’t know about the corn curls in our day or whatever, but the younger teachers now would have come up in that time when they used to eat them too!

While the food habits may be influenced by those within the inner setting, the access to unhealthy food is influenced by the availability of unhealthy options in both the inner and outer settings.

##### Economic challenges

The administrators indicated that in general, healthy foods were prohibitively expensive. While food providers were economically impacted when attempting to offer healthier options, parents also experienced economic tensions in trying to decide how to allocate household funds:

SS1: The canteen at one point in time tried to push a healthy environment business, it didn’t quite work, in that the children tended to avoid them, she was trying bags of fruit, and water and some other healthy options it didn’t quite work out for her financially because the children tended to avoid them [healthier foods].

PS2: Parents will look at their pocket and determine—despite all of the talking ‘bout obesity and diabetes and so on, all the heath challenges, the non-communicable diseases that are prevalent, a parent with four or five children, maybe even one, will sit down and determine […] how I and my child or my children going to survive today and tomorrow.

##### Vending

Food provision at schools is a significant financial enterprise which includes participation from vendors, canteen operators and the school staff. At the primary level, shops run by teachers and ancillary staff were identified as important means for schools to supplement their annual operational grants provided by government. At the secondary schools, fundraising was the domain of various clubs and societies. Regardless of which school, there seemed to be little consideration given to the quality of the food sold for raising funds as the focus is not on nutrition but on getting money quickly:

PP1: And this, what we’re doing now, I shan’t mention, is part of our own fundraising drive. Because when the grant comes from the government … the grant has to be spent in a particular way. And that [Internal Childhood Obesity Programming] is not...one of the ways. So we are on our own, sort of, to raise our own funds to help to implement some of the things we might want to do… So selling some things like what we are selling now will help you to make money quicker to spend. So it is a, kind of a two-edged sword.

School canteens operate under terms negotiated with their schools, and through that relationship, the most concerted effort was placed to ensure good nutrition on campus, compared to the other types of vending activities.

The concept of “vending” where an individual can sell treats to students on or near the environment of schools has been so canonized into the Barbadian national consciousness that attempts to disrupt this activity may result in public disapproval. Sanctioned vendors become institutionalized into a school’s community, and they typically have a strong relationship with school administrators and function as lay-staff.

SS2: They (vendors) operate a shift system. They sell on mornings before school and evenings after school. They are not open at lunch time. We share information with them, for example we give them a copy of the school’s calendar of events as it relates to early closure of school, so they know when the children will not be here. And that was an incentive for them cooperate with the school. They also give donations, monetary donations to school events, I would also say they assist with discipline within the school as well, because if they notice anything untoward, they will call the principal.

However, as independent operators, they have the least amount of accountability within the school’s organizational structure. Further, there are issues with unsanctioned vendors, persons who attempt to sell to students without the permission of school administrators. They, being unvetted, pose safety and security concerns. One of our interviews was interrupted by such unsanctioned vending, prompting administrators to report to the scene where food was being sold through the security fence. School administrators noted there are upsurges in the number of unsanctioned vendors during economic downturns.

SS4: There were about six or seven vehicles backing up and handing things over the fence. Now, tell me the truth, now let’s say you had two vendors that you knew and you could almost vouch for from a certain perspective of behaviour and knowing that they will not do anything untoward or anything like that. How do you, you got a brigade about seven now, you don’t know where they’re from! They just turn up! And handing things over the fence!... [We must] make sure that safety and security is, is maintained.

#### Networks and communications of the inner setting

The main parties of the school inner setting involved in the creation of nutrition programmes consists of school administrators, school staff and canteen operators (Fig. 1), with parents and students playing a passive role in nutrition related matters. No specific mechanisms to elicit the perspective of parents and students about school nutrition were reported, though administrators repeatedly spoke of taking their perspective into consideration. In order to implement these nutrition programmes in schools, the outer setting becomes integral. Schools have individually reached into the outer setting to work with several civil society organizations, faith-based organizations and private individuals in order to address nutritional needs identified at their institution.

PS1: The Muslim Association, before the Ministry had started the water project, the Muslim Association had donated a water cooler.

SS2: I also see a role for, resource persons coming in and speaking, I see nutritionists, at the level of (from) polyclinic, the hospital, coming in and speaking to students. I mean you do invite people to the school, who do come in and speak, but I can see there needs to be a real concerted effort in getting that done. I can see Heart and Stroke (Foundation) fanning out to different schools and asking for a little time to address students.

PS4: As I mentioned, outside agencies, I like the whole idea of school-community relations. ……….I should mention that we have, a gentleman. He is not a parent, but, I guess, he is affiliated with a Christian organisation and I think they’re, too, pushing, you know, sport and sports and healthy lifestyles.

It is clear that networks and communication for the inner and outer settings are entwined, and input from the outer setting is necessary in order for any school nutrition policy, guidelines or programming to be implemented.

#### Implementation climate of the inner setting

##### Readiness for change

All senior school administrators acknowledged childhood obesity to be a significant problem in the general Barbadian society. All but one agreed that students’ diets needed to be improved, and most had implemented independent interventions within their school in the last 2 years—not waiting on national policies but taking matters into their own hands—showing the urgency with which they regard the problem. School-level programmes enacted to address NCDs included school health fairs, cooking classes, community walks and water (only) days. Several resources for curriculum changes such as school gardens/farms and facilities for physical activity had already been present in schools for some time.

SS4: They used to actually have a farm here, you know, with animals, but I now trying to re re-establish it. As a matter of fact, I put a hundred and sixty-seven thousand dollars in the budget for it. I ain’t know if the Ministry going give me.

The administrators felt that these internal activities while not formally evaluated were having a positive impact on the children based on anecdotal information shared by teachers:

PS2: [O]ne of my colleagues, she has Infants [students aged 6-8], and she told me that sometimes when she does not remember the [Water] Wednesday she will sit down sometimes and see the children with the lunch and a bottle [of SSB], and she is wondering why these children are not drinking...the soft drinks, and then she would quickly remember. Or sometimes a child will say, ‘please teacher, today is Water Wednesday,’ and he bring to school a soft drink.

##### Relative priority

The urgency for implementation is relatively high but the following were viewed as competing priorities.

1. Limited resources: time, funds and personnel: insufficient subsidies provided by government for public schools affect the ability to implement new policies that needs additional resources.
2. Disciplinary issues in and near campuses: administrators reported that addressing violence occurring within the primary and secondary schools, as well as violence that students experience outside of school, but that affect their ability to fully participate in school, is a major priority.
3. Students challenged by undernutrition: school meal programmes are reported to be the sole source of meals for some primary school children during the week, and therefore, any food provided is accepted as the priority to for the students to have a meal.

As one administrator put it, “so there isn’t a whole lot of time that you have available within any particular week to focus on, food matters” (PS2), a focus on improving nutrition, despite the knowledge of improving educational outcomes for children, falls lower on the priority list, with these major competing issues at hand.

### Influences of the outer setting on policy development

Solutions to the challenges faced in the inner setting were seen to be inextricably linked to the outer setting. The METVT was identified as the chief stakeholder in the outer setting (Fig. 1), as administrators look to the government for guidance, to give authority to their activities, and to lead in the development of school nutrition polices. Mass media campaigns by governmental and non-governmental authorities in the outer setting have the potential to influence parental compliance to the MSNP.

#### External policies and incentives

Schools often benefitted from external policies, and incentives that were provided to them (as opposed to schools reaching out for assistance as described in the internal setting). Examples include health information disseminated by the Ministry of Health and Wellness on a population level through national mass media campaigns, and on an institutional level by HSFB facilitated programming in schools. These tended to improve parental engagement and student participation in healthy behaviours although schools are not necessarily directly engaged in the development of these external policies and directives:

PS2:…well sometimes you hear things on the radio, you know, that the Ministry is very concerned about, obesity and the diabetes and the hypertension within our schools. So yes, information is out there. But most of it I, like, read it in the media and so on?

Despite this, there was a perception that the population level health promotion activities as well as those within school curricula were responsible for students of all ages making modest improvements to their diets.

SS1: The message though has started to go through. I find a lot more children have been drinking water, we don’t know if that is because it’s healthy or I don’t know if it (is) because of money.

#### Cosmopolitan

This refers to cross-sector collaborations. Administrators acknowledged traces of inter-ministerial action between the METVT, the Ministries of Agriculture, Health and Wellness and Trade, in terms of provision of nutritious lunches to primary schools through the School Meals Department. However, they stated that higher level and stronger collaboration would be necessary for implementation of a mandatory school policy.

#### School needs and resources

Among the administrators, there was a general sense that the METVT, as recommended lead policy developers, would be well acquainted with the resources available at each school. Further, that government had limited financial and technical resources to lend to implementation, which made partnerships both within the inner and outer settings critical to the success.

### Summary of interconnectivity of the inner and outer settings

An assessment of the internal programming found that school administrators, school staff and canteen operators were the main parties involved in the creation of nutrition programmes (Fig. 1) in the inner setting with Civil Society and Corporate Entities from the outer setting assisting with the provision of resources (Loci 1 and 4). There seemed to be little participation of parents or students in the development process (loci 9 and 10). While the METVT was identified as the chief stakeholder in the outer setting, the administrators look to the government for guidance, to lend authority to their school-based activities, and for the development of appropriate policies for implementation. There also seemed to be little feedback (loci 3) to the METVT, on activities initiated at the local school inner setting. Civil society provides support both to METVT as well as directly to schools. The main nutrition relationship at loci 5 focused on a training programme which the METVT in collaboration with the Ministry of Health and Wellness coordinated for canteen operators. Some administrators mentioned that these programmes inspired their operators to initiate the process to establish healthier menus. However, they were unaware of any mechanism for operators to provide feedback to the METVT. Other Government ministries like the MHW communicate with schools only through the METVT. The current guidelines originate within the MHW and the disengagement of school administrators with these guidelines is explained by the lack of a direct communication channel between these entities.

## Discussion

Nutritional policies in schools are a national problem needing national solutions. This was a unanimous theme emerging from the perspective of school administrators who could only envision successful implementation of a school nutrition policy if the policy was initiated on a national (outer setting) rather than school level (inner setting), with all of society involvement in actioning implementation. This call for a macro-level leadership approach runs counter to some international experiences [22] but aligns closely with other experiences [23] that have found that the lack of macro-level direction could lead to confusion around how a policy is operationalized at the school level [23, 24]. The perceptions of the eventual implementers suggest that further attempts at internal programmes led by individual schools will continue to experience suboptimal effectiveness.

In examining the characteristics of the intervention, the documentary analysis showed that the current guidelines were moderately comprehensive with weak recommendations. Our participants who operate in the inner setting were disengaged from these guidelines and unaware of their content. The lack of engagement may be due to poor dissemination of the guidelines and a lack of engagement of the educational sector in their development. Face-to-face engagement has been shown to be one of the most effective dissemination strategy for policy makers and practitioners [25] but evaluation of a variety of strategies is necessary [26] and especially given movement and meeting restrictions in place as a result of the COVID-19 pandemic.

Administrators recognized that schools as environments were not insulated, many factors that affect behaviour within the school environment reside outside of that space. Mass media campaigns and civil society organization advocacy were noted to have positive effects on internal nutrition programmes within schools while negative models of unhealthy food consumption seen in the home and school environment threaten to negate sustained healthy student behaviour. Our findings of a need for intense collaboration among the actors within the school inner setting as well as between those in the inner and outer setting is in keeping with Latin American experiences which revealed through policy analysis that inter-sectoral partnerships and evidenced based advocacy by civil society were critical to successful childhood obesity policy implementation [21].

Addressing perceptions, in particular that a mandatory school nutrition policy will be economically burdensome, must be a priority [20]. School subventions from government are also perceived as insufficient and in need of supplementation. Schools’ internal programming has been frustrated by competition between the authorized food providers and itinerant vendors, leading to a return to the sale of unhealthy food.

### Implications for development of the MSNP

1. Given the resource limitations identified in the inner setting of the school, nutritionists from organizations of the outer setting will be needed to strengthen the content of the currently weak guidelines.
2. The development of a committee to advise on and monitor the strength of the language of the proposed MSNP has been used in other countries and is highly recommended given the findings of the documentary analysis and the fact that strength of the policy is a significant factor in successful policy implementation [27, 28].
3. Implementation must proceed on a whole-of-society approach (outer setting) not only at the school level. Gains made in the schools can easily be erased once students leave this inner setting, unless the rest of their environment is supportive.
4. Macro-level leadership (e.g. Ministry of Education) is highly recommended for the intended MSNP [23].
5. Civil society and faith-based organizations appear willing to support implementation of health school initiatives and should be engaged in the development, implementation and evaluation of the policy.
6. There is need for a licensing system for vendors to better regulate the food environment in and around schools. This would also provide the opportunity to more fully engage the vendors as stakeholders in order to build buy-in for the policy.

### Strengths and limitations

This study provided an in-depth focus on school administrators providing opportunity to truly understand the key issues of implementers. In using these voices, we provide strong representation for the school administrators whose leadership is key to implementation of the policy, and provide a foundation on which broader stakeholder engagement can now be built to gain wider perspectives on school policy implementation.

Our in-depth scrutiny of official policy documents guided by a well-used policy questionnaire is a strength which allows for standardization and international comparison in investigating this policy process. We chose the CFIR framework because our focus is on informing the implementation of the policy because it provided a broad scope from which to examine potential implementation issues. Several of the other frameworks focus on evaluation and were not as well suited for studying/informing the pre-implementation phase.

The lead author (NS) is a medical doctor and advocate for the implementation of the nutrition policy and thus care was taken in analysis meetings to consider that this author was more likely to highlight themes that facilitate implementation rather than highlight the barriers. The double coding of all transcripts, as well as use of multiple analysts including those external to the island, helped to negate the effect of this potential interaction.

### Conclusion

We present relationships between the inner and outer settings of the school environment that may affect the implementation of a school nutrition policy. Our findings have broad implications for improving the implementation of school nutrition policies using deterministic implementation frameworks like CFIR. This study provides rationale for government-led school nutrition policies with input from multiple ministries within government. Civil society organizations including faith-based organizations were also perceived as critical in this limited resource setting.

## Supplementary Information

**Additional file 1.** Interview Guide.

**Additional file 2.** Participant Information.

**Additional file 3: Supplemental Table 1**: Domains and Constructs which emerged as most relevant after thematic analysis with accompanying operationalized definition.

**Additional file 4.** COREQ Checklist.

## Data Availability

Given the small number of schools in Barbados, it is felt that some of the information if made public may make participants identifiable. For this reason, the datasets used and/or analysed during this study are available from the corresponding author on reasonable request.

## Abbreviations

CARICOM: Caribbean Community
CFIR: Consolidated Framework for Implementation Research
CSO: Civil Society Organization
HCC: Healthy Caribbean Coalition
HSFB: Heart and Stroke Foundation of Barbados
MHW: Ministry of Health and Wellness
METVT: Ministry of Education, Technological and Vocational Training
MSNP: Mandatory school nutrition policy
NSNG: National School Nutrition Guidelines
WELLSAT: Wellness School Assessment Tool
