# Supplementary material for: Understanding the need for a whole-of-society approach in school nutrition policy implementation: a qualitative analysis
Source: Implement Sci Commun. 2021 Jul 17;2:79. doi: 10.1186/s43058-021-00184-z (PMC8285724; doi:10.1186/s43058-021-00184-z)
Supplement: Supplementary file 1 — Additional file 1. Interview Guide. [file 43058_2021_184_MOESM1_ESM.docx]

# Appendix 1

## Qualitative Interview Guide

| General Opening Questions |
| --- |
| 1.Tell me a little about what activities the school has done to create a healthy school nutrition environment.  Prompt: If someone does not mention national nutrition guidelines then prompt to ask about the experience of the school’s management team in their awareness of and attempts Barbados national nutrition guidelines for schools. |
| 2. What are the most important aspects about addressing nutrition in schools? |
| Knowledge of guidelines |
| 1. In your opinion, what are some of the most important aspects of the guidance that is given on school nutrition? |
| 2. In your opinion, what are some of the least important aspects of the guidance that is given on school nutrition? |
| 3. Does the school have a nutrition officer assigned and what is his/her role? |
| Adaptability of implementation |
| 1. What kinds of changes or alterations do you think you will need to implement nutritional guidelines in your school? so it will work effectively in your setting? |
| 2. Who will decide or what is the process for deciding whether changes are needed to the intervention so that it works will in your setting? |
| 3. Are there components that should not be altered? |
| Complexity of guidelines implementation |
| 1. What are some of the challenges you have experience/or foresee in developing a school nutrition policy? |
| 2. What do you think can/should be done to help schools implement nutrition policies and guidelines? |
| Cost of implementation |
| 1. What resources do you anticipate will be needed/have been incurred in implementing nutrition guidelines? |
| 2. What resources were considered (including costs) when deciding to implement the guidelines? |
| 3. What support have you had in implementing the guidelines in your school? |
| Implementation planning |
| 1. What have you done (or what do you plan to do) to get a plan in place to have a health school nutrition environment? |
| 2. Can you describe the plan for implementing the healthy school nutrition environment? |
| 3. What role has your plan for implementation played during implementation (if applicable)? |
