# Supplementary material for: Understanding the need for a whole-of-society approach in school nutrition policy implementation: a qualitative analysis
Source: Implement Sci Commun. 2021 Jul 17;2:79. doi: 10.1186/s43058-021-00184-z (PMC8285724; doi:10.1186/s43058-021-00184-z)
Supplement: Supplementary file 2 — Additional file 2. Participant Information. [file 43058_2021_184_MOESM2_ESM.pdf]

THE UNIVERSITY OF THE WEST INDIES
AT CAVE HILL, BARBADOS

School environment case studies

Participant information sheet (Principals/School Management)

We would like to invite you to take part in a research study to explore the experiences of school management with the implementation of a healthy school policy initiative on the food environment and to describe the nature of the internal and external food environments around primary and secondary schools in Barbados. We would like to conduct a semi-structured interview to explore school managers thoughts about the support, costs and challenges regarding implementation of the healthy lifestyle guidelines released by the Ministries of Health and Education. To help you decide if this study is of interest to you, please read the following information carefully about what your participation would involve.

What is the purpose of the study?

Our main aim is to discover the facilitators and barriers of implementation of school food guidelines by speaking with school managers and those tasked with implementing the guidelines about their experiences. We hope that by identifying challenges and hearing about what has facilitated the process, this will enable policy makers to improve the penetration and suitability of future guidelines/policies/regulations. Secondarily we wish to describe the school food environment at select primary and secondary schools in Barbados to provide us with a baseline for the current status of our schools.

Why have I been invited?

As a leader within your school you have been chosen as one whose overall task it is to ensure the implementation of the guidelines.

Do I have to take part?

It is up to you to decide whether or not to take part. If you decide to take part, you are free to stop taking part at any time during the research without giving a reason.

What will happen to me if we take part?

If you agree to take part, we will arrange an appointment for our researcher to meet you and conduct the interview. This can happen during or before/after office hours. We expect the interview to last for about one hour, but if you are willing it could last up to 90 minutes.

The researcher will make an audio recording of the interview. The recording will be transcribed verbatim. The person transcribing the recording will not be able to identify you from the recording. The recording and the transcript will then be stored securely at the George Alleyne Chronis Disease Research Centre a unit of the University of the West Indies Cave Hill.

After the initial interview of all participants, responses will be collated and reported only in aggregate form. The themes emerging from these interviews will be used in a journal article for research purposes.

What are the possible disadvantages of taking part?

There is no disadvantage to you except that if you do decide to take part, you will need to give up about one hour of your time for the interview.

What are the possible benefits of taking part?

The information collected during this study will give us a better understanding of the complexities related to implementing healthy lifestyle guidelines in the school setting.

Will my taking part in this study be kept confidential?

All information that is collected from you during the course of the research will be kept strictly confidential and stored securely at the George Alleyne Chronic Disease Research Center a unit of the University of the West Indies Cave Hill. You will be identified by an unique project number and any information obtained from you will have your name and address removed so that you cannot be recognised from it.

What will happen if we don't want to carry on with the study?

You are free to withdraw from the study at any time and without giving a reason. If you decide to withdraw, or if you are no longer able to take part in the study, we will use the data collected up to the time of your withdrawal.

What will happen to the results of the research study?

We expect to publish the results of the study in the next few years. We may use short excerpts of what you say to illustrate the findings, but we will not publish any details that identify you personally. We can send you a summary of the overall results.

Who is organising and funding the research?

This study is organised by the George Alleyne Chronic Disease Research Center at the University of the West Indies Cave Hill and approved by the Barbados Ministry of Health. Funding will be provided by Bloomberg Philanthropies.

Who has reviewed the study?

This study has been reviewed and given a favourable opinion by the Institutional Review Board (IRB) of the University of the West Indies and the Barbados Ministry of Health to protect your safety, rights, well-being and dignity.

Who can we contact for further information?

If you would like further information, please contact the main researcher of the study, Dr. Natasha Sobers, by email at natasha.sobers@cavehill.uwi.edu or by telephone on 240-0313.

Natasha Sobers, MBBS, MPH, FRSPH, PhD
Principal Investigator, Lecturer Public Health and Epidemiology

Questions or concerns about the ethics of this study can be directed to the University of the West Indies-Cave Hill/Barbados Ministry of Health Research Ethics Committee/Institutional Review Board, Office of Research, UWI - Cave Hill, Ms. Kristina Bryant kristina.bryant@cavehill.uwi.edu
