# Supplementary material for: Understanding the need for a whole-of-society approach in school nutrition policy implementation: a qualitative analysis
Source: Implement Sci Commun. 2021 Jul 17;2:79. doi: 10.1186/s43058-021-00184-z (PMC8285724; doi:10.1186/s43058-021-00184-z)
Supplement: Supplementary file 3 — Additional file 3: Supplemental Table 1: Domains and Constructs which emerged as most relevant after thematic analysis with accompanying operationalized definition. [file 43058_2021_184_MOESM3_ESM.docx]

Appendix 3: Supplemental Table 1

Supplemental Table 1: Domains and Constructs which emerged as most relevant after thematic analysis with accompanying operationalized definition

| Domain | Constructs Under Study | Theoretical Definition | Operationalised Definition |
| --- | --- | --- | --- |
| Characteristics of the Intervention: Given that most school administrators were unaware of the current guidelines we considered how the constructs may inform the development of a new mandatory school nutrition policy | Intervention Source | Pertains to whether stakeholders believe the policy was developed internally or externally, and from that the legitimacy of the policy. | We considered whether the school administrators believed the policy should be developed at the level of the school (inner setting) or the Ministry of Education which is a government body in the outer setting |
|  | Complexity | Refers to the perceived difficulty stakeholders identify related to implementation. | Threats to policy implementation identified by school administrators |
|  | Cost | Related to the cost of the implementing the policy inclusive of investment, supply and opportunity costs. | Opportunity costs associated with implementation. |
|  |  |  |  |
| Inner Setting: Internal was defined as the school. This included activities pertaining to students, parents, teachers, canteen operators and school vendors | Structural characteristics | Social architecture of the organisation, it’s age, maturity, and size. | Number of students in participating schools, number of teachers, Presence of canteens and school meals service |
|  | Networks and Communication | The nature and quality of social networks, and communication within of the organisation involved. | Partnerships schools have developed in the process of developing a healthier food environment. |
|  | Culture | Norms, values, and basic assumptions of a given organization | The norms, values operating at the level of the school |
|  | Implementation Climate  *Tension for change:*  *Relative Priority:* | The organisations readiness for change |  |
|  |  | The degree to which stakeholders perceive the current situation is intolerable. | School administrators’ perceptions about the need to improve quality of food students ate. Competing priorities were documented as reported. |
|  |  | The implementers perception of the importance of implementing the policy. |  |
| Outer Setting: External was defined as organisations other than schools. These included Ministry of Education, Ministry of Health and Wellness, other government ministries, Local Civil Society, and International Organisations | Patient needs & resources | Related to the extent needs of the patient and the barriers and facilitators associated with their role in the policy are known by the policy makers. | School administrators’ perception on whether policy makers are aware of their needs and the needs and resources of the school particularly related to implementation of National School Nutritional Guidelines |
|  | Cosmopolitanism | Refers to the degree to which an organisation is networked to other external organisations. | The strategic partnerships the Ministry of Education developed to facilitate the implementation of National School Nutritional Guidelines |
|  | External Policies and Incentives | External policies which aid in the spread of the policies. | Policies of organisations and ministries other than MOE, that school administrators perceive as facilitating implementation |
